# Non-affective psychotic disorders and risk of dementia: a systematic review and meta-analysis

**DOI:** 10.1017/S0033291722002781

**Published:** 2022-11

**Authors:** Sara El Miniawi, Vasiliki Orgeta, Jean Stafford

**Affiliations:** 1 Division of Psychiatry, University College London (UCL), London, UK; 2 MRC Unit for Lifelong Health and Ageing, UCL, London, UK

**Keywords:** Dementia, Meta-analysis, Non-affective psychotic disorders, Risk, Systematic review

## Abstract

Non-affective psychotic disorders have been associated with an increased risk of developing dementia. However, research in this area remains limited, highlighting the need for an up-to-date systematic review and meta-analysis of the evidence. We aimed to systematically review and quantify the risk of dementia associated with psychotic disorders. We searched four electronic databases for longitudinal studies investigating non-affective psychotic disorders and subsequent dementia. We used random-effects meta-analyses to pool estimates across studies and assessed risk of bias for each study. Non-affective psychotic disorders were associated with increased risk of all-cause dementia; pooled risk ratio (RR) = 2.52, 95% confidence interval (CI) (1.67–3.80), *I*^2^ = 99.7%, *n* = 12,997,101; 11 studies, with high heterogeneity between studies. Subgroup analyses indicated stronger associations in studies with shorter follow-up periods, conducted in non-European countries, published after 2020, and where ≥60% of the sample were female. The risk was higher in people aged <60 years at baseline, in typical and late-onset psychotic disorders versus very late-onset psychosis, in broader psychotic disorders vs schizophrenia, and in prospective vs retrospective studies. Associations remained after excluding low quality studies (pooled RR = 2.50, 95% CI (1.71–3.68), *I*^2^ = 99.0%). Our review finds a substantial association between psychotic disorders and subsequent dementia. Our findings indicate that psychotic disorders are a potentially modifiable risk factor for dementia and suggest that individuals with psychotic disorders need to be closely monitored for cognitive decline in later life. Further research is needed to investigate the mechanisms underlying the association between psychotic disorders and dementia.

## Background

Dementia is a syndrome characterised by progressive cognitive and functional decline constituting one of the leading causes of disability worldwide (Duong, Patel, & Chang, 2017; World Health Organization [WHO], 2017). The prevalence of dementia is estimated to double every 20 years, making it a major public health concern, with estimated worldwide costs reaching $1 trillion per year in 2018 (Wimo et al., 2017; Ferri et al., 2005; Prince et al., 2015). Several psychiatric disorders have been found to increase risk of developing dementia, with depression, anxiety, and post-traumatic stress disorder (PTSD) being among those most extensively studied. There is also increasing research interest in the relationship between non-affective psychotic disorders and future dementia risk. Schizophrenia and related non-affective psychotic disorders such as schizoaffective disorder, and delusional disorder are commonly characterised by symptoms such as hallucinations and delusions, a lack of motivation, and impairments in cognitive functioning (Arciniegas, 2015). Cognitive impairment is a core symptom occurring both in dementia and psychotic disorders, although the underlying cause and course of cognitive impairment in psychotic disorders remains unclear (Alkan, Davies, & Evans, 2021; Kahn, 2019). Understanding whether psychotic disorders represent a risk factor for dementia is important for its prevention and timely treatment and could lead to improved clinical management and a better understanding of the nature of cognitive impairment in psychotic disorders (Livingston et al., 2020).

Despite increasing research interest in the association between psychotic disorders and subsequent cognitive decline and dementia, research in this area remains relatively sparse. Although a previous meta-analysis found an increased risk of dementia in people with schizophrenia, only six studies were identified, with heterogeneous designs (Cai & Huang, 2018). Since the publication of the previous review, multiple large longitudinal studies have been published examining the association between psychotic disorders and subsequent dementia.

In addition, little is known about whether dementia risk differs for people with late-onset (LOS) and very late-onset schizophrenia-like psychoses (VLOSLP), which have symptom onset after age 40 and 60 years respectively, relative to psychotic disorders with a more typical age-at-onset, occurring in late adolescence and early adulthood (Howard, Rabins, Seeman, Jeste, & the International Late-Onset Schizophrenia Group, 2000; Kessler et al., 2007). Differences in epidemiology and presentation have been found in those with LOS and VLOSLP, including a higher prevalence among women and fewer negative symptoms (Howard et al., 2000; Stafford, Howard, & Kirkbride, 2018; Stafford, Howard, Dalman, & Kirkbride, 2019; Vahia et al., 2010). Given these differences, LOS and VLOSLP might be expected to show different patterns and strength of association with dementia compared to psychotic disorders with a more typical age-at-onset.

Furthermore, given that dementia is commonly preceded by neuropsychiatric symptoms, which can include hallucinations and delusions (Wise, Rosenberg, Lyketsos, & Leoutsakos, 2019), examining variation in associations by psychotic disorder age-at-onset and duration between psychotic disorders and dementia could provide insight into whether associations are likely to be causal, or to reflect reverse causation due to neuropsychiatric symptoms as part of the dementia prodrome. For example, stronger associations with dementia for LOS and VLOSLP, and when the duration between psychotic disorders and dementia is shorter, would be more indicative of reverse causation, whereas longstanding associations would be more consistent with a causal relationship.

A comprehensive and up-to-date systematic review and meta-analysis is therefore now required in order to provide an accurate quantitative estimate of the association between non-affective psychotic disorders and future risk of dementia, with a particular focus on investigating potential underlying mechanisms and for updating current life course models of dementia prevention.

To address gaps in previous knowledge, this systematic review and meta-analysis aimed to investigate whether non-affective psychotic disorders are longitudinally associated with an increased risk of dementia and to provide an up-to-date estimate of the association. In addition, we aimed to compare differences in association among those with LOS and VLOSLP relative to psychotic disorders with a more typical age-at-onset. We additionally examined whether other non-affective psychotic disorder diagnoses, such as delusional disorder, in addition to schizophrenia, are associated with an increased risk of dementia. To further investigate potential sources of heterogeneity, we examined whether variables such as sex, study type, study quality, follow-up study interval, and anti-psychotic medication use influenced results.

## Method

We followed current PRISMA guidelines (Page et al., 2021) for the reporting of systematic reviews (online Supplementary Table S1) and registered our review with PROSPERO: CRD42021255719.

### Search strategy

Four databases, Medline, Embase, PsycInfo, and CINAHL plus, were searched for published literature and two additional databases, Open Grey and Ethos, were searched for grey literature up to 29th of June 2021. We re-ran the search using Medline until March 2022 to check for recent papers. The search terms used were broadly related to non-affective psychotic disorders, dementia, and type of study (see online Supplementary Table S2 for details of search terms used). Full-texts were searched for all identified studies, and two authors independently screened all full-text articles for relevant studies. Disagreements were discussed with a third reviewer. Reference lists of all relevant studies reaching the full-text phase were hand searched to ensure no relevant studies were missed.

### Selection criteria

We included both prospective and retrospective longitudinal studies examining the association between psychotic disorders and dementia. The population of interest were adults aged ⩾18 years with a clinical diagnosis of a non-affective psychotic disorder (ICD-11, 6A20-6A2Z or equivalent), and the comparison group were adults without a non-affective psychotic disorder (American Psychological Association [APA], 2013; WHO, 2018). Dementia diagnosis was also based on clinical criteria (ICD-11, 8A20-8A2Z, NINCDS-ADRA or equivalent) (APA, 2013; Blacker et al., 1994; WHO, 2018).

### Data extraction

Data were extracted independently by two authors and included: name of authors, year of publication, setting, sample size, study type, assessment/diagnostic criteria of psychotic disorder and dementia, length of follow-up, relevant effect estimates, risk of bias assessment, and control of confounders. Hazard ratios (HRs), risk ratios (RR), odds ratios (ORs), incidence rate ratios (IRRs), standardised mortality ratios (SMRs), and standardised incidence ratios (SIRs), along with standard errors and confidence intervals (CIs), were extracted for the meta-analysis. We combined various measures of relative risk in line with current guidelines, and the rare disease assumption, which states that various measures of relative risk will approximate each other when the outcome investigated is sufficiently rare (Greenland, 1987).

### Risk of bias assessment

Two reviewers independently assessed risk of bias in all included studies using a modified version of the Newcastle-Ottawa Scale (NOS) (see online Supplementary Table S3), covering three risks of bias domains: selection of study groups, comparability and study design, and outcome (Stang, 2010). Disagreements were resolved with a third reviewer. Small-study effects were assessed using Egger's weighted regression and visual inspection of a funnel plot (Lin & Chu, 2018).

### Data analysis

STATA (Version 17.1) and the ‘metan’ and ‘metareg’ commands were used to conduct the meta-analyses and meta-regressions. The generic inverse variance method and a random-effects model was used to obtain pooled relative risk estimates across studies that reported IRRs, HRs, RRs, ORs, SMRs, and SIRs. Heterogeneity was measured using the χ^2^ Cochran's *Q* test and the *I*^2^-statistic. We examined sources of heterogeneity using subgroup analyses, sensitivity analyses, and meta-regression.

## Results

### Study selection

**Fig. 1.**
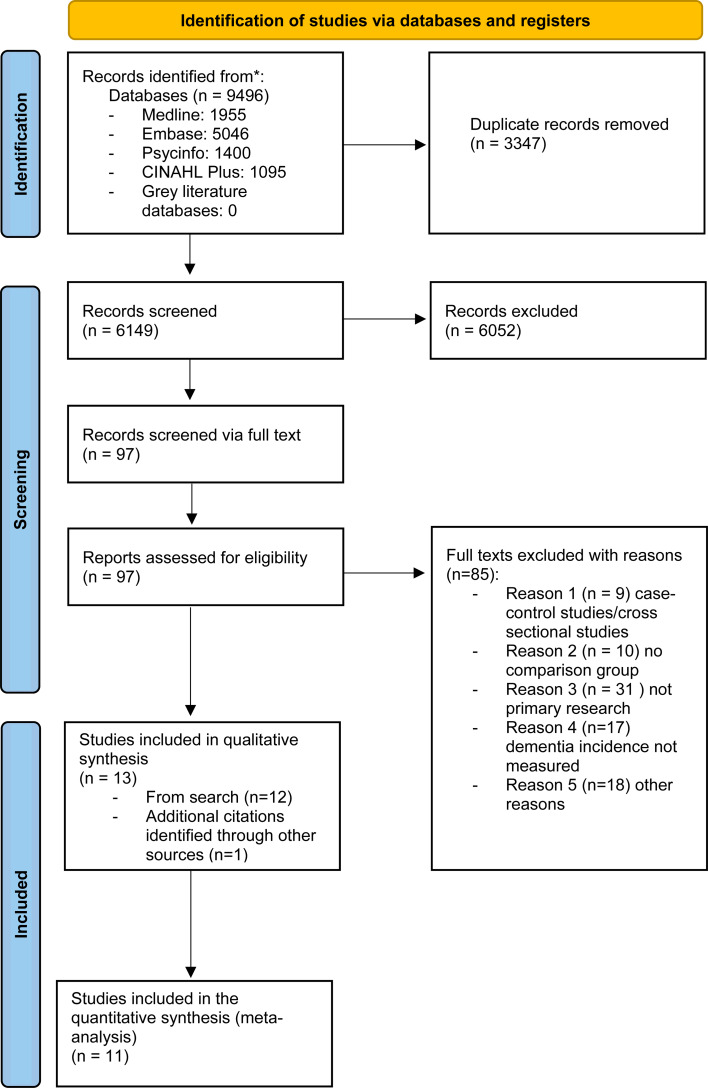
PRISMA flow diagram of the study selection process.

The study selection process followed the Preferred Reporting Items for Systematic Reviews and Meta-analysis (PRISMA) (Page et al., 2021). A total of 9496 studies were identified through the search process, with a total of 6149 studies remaining after removal of duplicates (Fig. 1). Following the initial screening of titles and abstracts, a total of 73 studies remained, which were screened for eligibility. When we re-ran our search to update it, an additional 24 studies were identified that were eligible for full-text screening. Of all the full-text studies identified 85 were excluded due to not meeting eligibility criteria (see online Supplementary Table S4), leaving 12 studies meeting our inclusion criteria. One newly published study was identified after the initial screening (Richmond-Rakerd, D'Souza, Milne, Caspi, & Moffitt, 2022), resulting in a total of 13 included studies of which 11 were eligible to be included in the meta-analysis. Three of the identified studies had the same comparison population, but two of these focused specifically on late-onset acute and transient psychosis (LOATP) and late-onset delusional disorder (LODD) (Kørner, Lopez, Lauritzen, Andersen, & Kessing, 2008, 2009a, 2009b). These two studies were not included in the meta-analysis but were retained in the narrative synthesis, given that no other studies examined these specific disorders.

### Study characteristics

Information on study characteristics is presented in Table 1. Of the 13 included studies, three were prospective and 10 were retrospective. Studies were published between 2003 and 2022, with four studies published between 2003 and 2014, four published between 2015 and 2019, and five published in 2020 or after. Sample sizes across studies ranged from 61 to 8 011 773 participants, with a total of 12 997 101 participants. Study follow-up periods ranged from 1.57 to 33 years (median: 11 years). Studies were conducted in Europe (Denmark, Finland, Sweden, and the United Kingdom), the United States, Australia, Taiwan, New Zealand, and Israel.

**Table 1.** Characteristics of included studies

Author, country, year of publication	Study type	Sample at baseline, age minimum or age range	% Female	Participant recruitment	Follow-up (years)	Assessment/diagnostic criteria (for dementia and psychotic disorder)	Effect estimate, after follow-up^a^	Variables adjusted for	Quality rating
Almeida et al. (2018) Australia	Prospective	*N* = 37 770 Age: 65–85	Females: 0%	Australian electoral roll	17.7	Psychotic disorders: ICD-8/9/10 Dementia: ICD-8/9/10	HR: 2.57; 95% CI (2.23–2.96)	HR adjusted for age, cardiovascular diseases, cancer, chronic respiratory diseases, gastrointestinal and renal diseases, hearing loss, alcohol use disorders, depressive and bipolar disorder	Good
Brodaty et al. (2003) Australia	Prospective	*N* = 61 Age: 50–87	Females in schizophrenia cohort: 78.9%	Patients recruited from local mental health services	5	Schizophrenia: DSM-IV Dementia: DSM-IV	OR: 16.52; 95% CI (1.85–147.8)	OR unadjusted	Poor
Kershenbaum et al. (2020) UK	Retrospective	*N* = 28 340 Age: 65+	Females in schizophrenia cohort: 57.7%	Electronic health records of people referred to Cambridge and Peterborough NHS Foundation Trust	6	Schizophrenia: ICD-10 Dementia: ICD-10	SIR: 2.9; 95% CI (1.5–4.7)	SIR unadjusted	Fair
Kodesh et al. (2020) Israel	Prospective	*N* = 94 120 Age: 60–90	Females in schizophrenia cohort: 62.6%	Health Maintenance Organisation data	4 years and 10 months	Psychotic disorders: ICD-8/9 Dementia: ICD-8/9/10	HR: 2.89; 95% CI (2.15–3.89)	HR adjusted for year born, sex, smoking status in 2012, antipsychotic medication exposure, adverse reactions following antipsychotic medication, medical comorbidities	Good
Kørner et al. (2008) Delusional disorder, Denmark^b^	Retrospective	*N* = 8739 Age: 60+	Females in delusional disorder cohort: 77.6%	Danish population register data	Median: 1.87	Delusional disorder: ICD-10 Dementia: ICD-10	IRR: 5.49, 95% CI (4.81–6.26)	IRR adjusted for age at first contact, diagnosis of substance abuse and calendar time	Good
Kørner et al. (2009b), LOS and VLOSLP, Denmark	Retrospective	*N* = 12 616 (for 40-year cut-off) *N* = 7712 (for 60-year cut-off) Age: 40+	Females in LOS cohort: 47.9%	Danish population register data	4.58	Schizophrenia: ICD-10 Dementia: ICD-10	LOS SMR: 2.36, 95% CI (1.54–3.62) VLOSLP SMR: 2.21; 95% CI (1.39–3.5)	SMR matched for age, sex, and calendar time	Good
Kørner et al. (2009a), Acute and transient psychotic disorders, Denmark^b^	Retrospective	*N* = 8062 Age: 60+	Females in acute and transient cohort: 67.5%	Danish population register data	Median: 1.57 years	Acute and transient psychotic disorder: ICD-10 Dementia: ICD-10	SMR: 8.12; 95% CI (6.7–9.74)	SMR matched for age, sex, and calendar time	Good
Lin et al. (2018) Taiwan	Retrospective	*N* = 30 200 Age: 18+	Females in schizophrenia cohort: 43.3%	Taiwan National Health Insurance Research Database	14	Schizophrenia: ICD-9-CM Dementia: ICD-9-CM	HR: 2.01; 95% CI (1.42–2.59)	HR adjusted for sex, age, medical comorbidities, medications	Good
Ribe et al. (2015) Denmark	Retrospective	*N* = 2 845 440 Age: 50+	Females in schizophrenia cohort: –	Danish population register data	18	Schizophrenia: ICD-8/9 Dementia: ICD-8/9/10	IRR: 2.13; 95% CI (2.00–2.27)	IRR adjusted for age, sex, calendar period	Good
Richmond-Rakerd et al. (2022) New Zealand	Retrospective	*N* = 1 711 386 Age: 21–60	Females in psychotic disorder cohort: –	New Zealand Integrated Data Infrastructure provided information on public hospital records	30	Psychotic disorders: ICD-9/10 Dementia: ICD-9/10	RR: 6.20; 95% CI (5.67–6.78)	RR adjusted for sex, birth year, and pre-existing chronic physical disease diagnosis	Good
Stafford et al. (2021) Sweden	Retrospective	*N* = 169 499 Age: 60+	Females in VLOSLP cohort: 60.56%	Swedish population register data	30	VLOSLP: ICD-8/9/10 Dementia: ICD-8/9/10	HR: 4.22, 95% CI (4.05–4.41)	HR adjusted for age, sex, region of birth, disposable income at age 60, education	Good
Stroup et al. (2021) USA	Retrospective	*N* = 8 011 773 Age: 66+	Females in schizophrenia cohort: 56.6%	Medicare beneficiaries, USA, register-based	11	Schizophrenia: ICD-8/9/10 Dementia: ICD-8/9/10	IRR: 1.47; 95% CI (1.45–1.5)	Unadjusted IRR	Fair
Tapiainen et al. (2017) Finland	Retrospective (nested case-control)	*N* = 55 896 Av. age: 79.7	Females in psychotic disorder cohort: –	Finish clinical registry of Alzheimer's disease	33	Psychotic disorders: ICD-10 Alzheimer's: NINCDS-ADRDA and DSM-IV	5-year window OR: 0.94; 95% CI (0.81–1.10) 10-year window OR: 0.87; 95% CI (0.73–1.02)	OR matched for age, sex. OR adjusted for Charlson comorbidity index and drug use	Good

*Note.* ICD-8/9/10, International Classification of Diseases, 8th/9th/10th revision; OR, odds ratio; HR, hazard ratio; SIR, standardised incidence ratio; RR, risk ratio; IRR, incidence rate ratio; CI, confidence interval; DSM-IV, Diagnostic and Statistical Manual of Mental Disorders, 4th Revision; NINCDS-ADRDA, National Institute of Neurological and Communicative Disorders and Stroke-Alzheimer's Disease and Related Disorders; Charlson comorbidity index: predicts mortality from a range of medical comorbidities (diabetes, liver disease, heart failure, etc.). *N*, number of participants; –, data not reported.

a If not stated otherwise, effect estimate of dementia incidence in non-affective psychotic disorder compared to general population.

^b^ Not included in the meta-analyses.

Study participants were aged 18 years and above, with most being over the age of 60. The percentage of females across studies ranged from 0 in an only male cohort to 78% (Almeida et al., 2018). Studies focused on a range of non-affective psychotic disorder diagnoses including LOATP (*n* = 1; age-at-onset ⩾ 60) (Kørner, Lopez, Lauritzen, Andersen, & Kessing, 2009a), LODD (*n* = 1; age-at-onset ⩾ 60) (Kørner et al., 2008), LOS and/or VLOSLP (*n* = 5; age-at-onset ⩾ 40, or >60) (Almeida et al., 2018; Brodaty, Sachdev, Koschera, Monk, & Cullen, 2003; Kodesh et al., 2020; Kørner, Lopez, Lauritzen, Andersen, & Kessing, 2009b; Stafford et al., 2021), schizophrenia (*n* = 1) (Lin, Chung, Chen, & Chi, 2018), psychotic disorders (*n* = 2) (Richmond-Rakerd et al., 2022; Tapiainen, Hartikainen, Taipale, Tiihonen, & Tolppanen, 2017), and schizophrenia in older people (*n* = 3) (Kershenbaum et al., 2020; Ribe et al., 2015; Stroup et al., 2021). Most studies investigated incidence of all-cause dementia, with one study focusing on incidence of Alzheimer's disease (Tapiainen et al., 2017).

### Characteristics of the sample, exposure, and outcome across studies

Nine studies used population registers with records of clinical diagnoses of psychotic disorders and dementia based on ICD-8, -9, or -10 (Almeida et al., 2018; Kodesh et al., 2020; Kørner et al., 2008, 2009a, 2009b; Ribe et al., 2015; Richmond-Rakerd et al., 2022; Stafford et al., 2021; Tapiainen et al., 2017). One study used medical insurance data (Stroup et al., 2021) and three studies used data from mental health services, where both psychotic disorders and dementia were clinically diagnosed using ICD-9 or ICD-10, or DSM-III and DSM-IV criteria (Brodaty et al., 2003; Kershenbaum et al., 2020; Lin et al., 2018).

### Risk of bias and adjustment for confounders

Ten studies scored in the higher range of methodological quality, two studies (Kershenbaum et al., 2020; Stroup et al., 2021) were rated as fair, and one as poor (Brodaty et al., 2003) (Table 2). Almost all studies accounted for basic sociodemographic confounders such as age and sex (see Table 1). Several studies adjusted for comorbidities (Almeida et al., 2018; Lin et al., 2018; Ribe et al., 2015; Richmond-Rakerd et al., 2022; Tapiainen et al., 2017), alcohol or substance use disorders (Almeida et al., 2018; Ribe et al., 2015; Tapiainen et al., 2017), medications (Kodesh et al., 2020; Lin et al., 2018), smoking status (Kodesh et al., 2020), and income and education level (Stafford et al., 2021).

**Table 2.** Results of NOS assessment for study quality

	Selection				Comparability		Outcome		
	Representativeness	Control group	Ascertainment of exposure	Outcome not at baseline	Adjusted covariates^a^	Study type	Assessment of outcome	Follow-up^b^	Overall quality
Almeida et al. (2018)	1	1	1	1	1	1	1	1	Good
Brodaty et al. (2003)	0	0	1	0	0	1	1	1	Poor
Kershenbaum et al. (2020)	1	0	1	0	0	0	1	2	Fair
Kodesh et al. (2020)	1	1	1	1	1	1	1	1	Good
Kørner et al. (2009a)	1	1	1	1	1	0	1	1	Good
Kørner et al. (2009b)	1	1	1	1	1	0	1	1	Good
Kørner et al. (2008)	1	1	1	1	1	0	1	1	Good
Lin et al. (2018)	1	1	1	1	1	0	1	2	Good
Ribe et al. (2015)	1	1	1	1	1	0	1	2	Good
Richmond-Rakerd et al. (2022)	1	1	1	1	1	0	1	2	Good
Stafford et al. (2021)	1	1	1	1	1	0	1	2	Good
Stroup et al. (2021)	1	1	1	1	0	0	1	2	Fair
Tapiainen et al. (2017)	1	1	1	1	1	0	1	2	Good

a Adjusted for at least two covariates.

b Up to two points for follow-up.

### Primary meta-analysis of psychotic disorders and risk of dementia

**Fig. 2.**
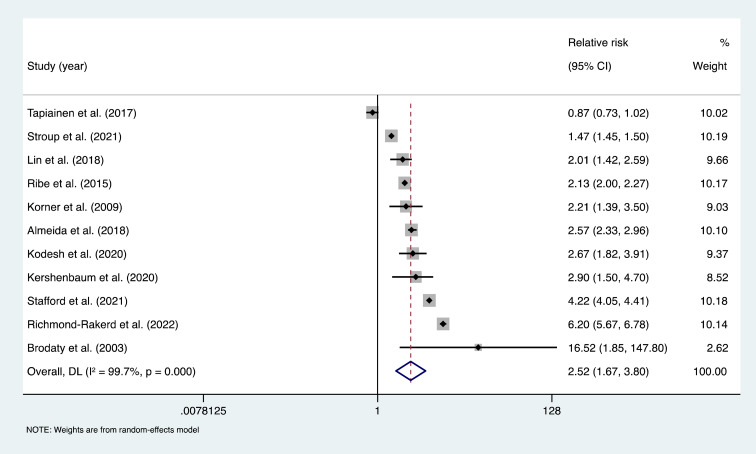
Meta-analysis of effect estimates of non-affective psychotic disorders compared to no non-affective psychotic disorders on risk of dementia.

Pooling estimates from 11 studies showed that psychotic disorders were associated with an increased risk of all-cause dementia; pooled RR = 2.52, 95% CI (1.67–3.80), *I*^2^ = 99.7%, *p* < 0.001; a total of 12 997 101 participants and around 119 274 individuals with psychotic disorders; median follow-up of 11 years (Fig. 2). For the Tapiainen et al. (2017) study, we used the estimated risk based on the 10-year time interval, given that it was the longest period reported. For the Kørner et al. (2009b) study we used the estimate for LOS, and for the Stroup et al. (2021) study we calculated an IRR based on the incidence rate data, which was provided separately for those with and without schizophrenia. Of the 11 studies included, 10 reported that psychotic disorders increased risk of dementia, while one study by Tapiainen et al. (2017) reported no significant association.

The Brodaty et al. (2003) study was excluded from the funnel plot for small-study effects, due to being an outlier that influenced the scale of the plot (see online Supplementary Fig. S1). Visual inspection of the funnel plot indicated some asymmetry, however the Egger's test did not provide evidence of small-study effects (*t* = 1.22, *p* = 0.252) (Ioannidis & Trikalinos, 2007).

### Subgroup analyses and meta-regression

Our subgroup analyses indicated that the risk of dementia was higher in individuals with typical and late-onset psychotic disorders (onset <40 years and >40 years) compared to individuals with VLOSLP (onset >60 years); pooled RR = 3.10, 95% CI (2.33–4.14), *I*^2^ = 77.5%, *p* = .0.004, *v.* pooled RR = 2.77, 95% CI (1.74–4.40), *I*^2^ = 98.9%, *p* < 0.001, *n* = 3). Studies with a follow-up of <10 years showed a larger effect compared to studies with a follow-up of ⩾10 years; pooled RR = 2.64, 95% CI (1.98–3.51), *I*^2^ = 10.9%, *p* = 0.338, *v.* pooled RR = 2.33, 95% CI (1.43–3.82), *I*^2^ = 99.8%, *p* < 0.001. Risk was higher in studies conducted in non-European countries compared to those recruiting in European countries; pooled RR = 2.96, 95% CI (1.56–5.62), *I*^2^ = 99.4%, *p* < 0.001, *v.* pooled RR = 2.04, 95% CI (1.12–3.71), *I*^2^ = 99.5%, *p* < 0.001. Studies in which ⩾60% of the sample were female showed a higher risk compared to studies with less than <60% of the sample being female; pooled RR = 3.24, 95% CI (2.09–5.02), *I*^2^ = 79.0%, *p* = 0.003, *v.* pooled RR = 2.10, 95% CI (1.42–3.12), *I*^2^ = 96.7%, *p* < 0.001. In addition, the association between psychotic disorders and dementia was stronger in studies published in 2020 or after, relative to those published before 2020; pooled RR = 3.13, 95% CI (1.55–6.33), *I*^2^ = 99.9%, *p* < 0.001, *v.* pooled RR = 1.93, 95% CI (1.34–2.77), *I*^2^ = 95.8%, *p* < 0.001.

Studies investigating broader non-affective psychotic disorders showed a higher risk compared to studies investigating schizophrenia; pooled RR = 2.78, 95% CI (1.57–4.91), *I*^2^ = 99.4%, *p* < 0.001, *v.* pooled RR = 2.13, 95% CI (1.66–2.73), *I*^2^ = 95.9%, *p* < 0.001. Risk was higher in prospective studies compared to retrospective studies; pooled RR = 2.65, 95% CI (2.10–3.35), *I*^2^ = 28.3%, *p* = 0.248, *v.* pooled RR = 2.34, 95% CI (1.45–3.78), *I*^2^ = 99.8%, *p* < 0.001. Our final set of analyses showed that studies in individuals with a minimum age <60 at baseline showed a higher risk of dementia compared to studies with a minimum age ⩾60; pooled RR = 3.16, 95% CI (1.59–6.28), *I*^2^ = 99.8%, *p* < 0.001, *v.* pooled RR = 2.61, 95% CI (1.39–4.92), *I*^2^ = 99.8%, *p* < 0.001. Correlations between potential moderators are reported in online Supplementary Table S5.

In line with current recommendations, we conducted meta-regressions for characteristics that were examined in at least 10 studies (Higgins et al., 2019). We did not find evidence that heterogeneity between estimates was explained by type of psychotic disorder (meta-regression *p* = 0.66), follow-up time-point (*p* = 0.59), geographical region (*p* = 0.41), or year of study publication (*p* = 0.22) (see online Supplementary Table S6).

### Sensitivity analysis

Sensitivity analyses showed that the strength and direction of the results, and between study heterogeneity (*I*^2^ range: 96.7–99.6) remained the same after excluding any single study. Excluding low-quality studies (*n* = 3) (Brodaty et al., 2003; Kershenbaum et al., 2020; Stroup et al., 2021) and those not adjusting for at least two confounders (*n* = 3) (Brodaty et al., 2003; Kershenbaum et al., 2020; Stroup et al., 2021) did not influence the pooled estimate; pooled RR = 2.50, 95% CI (1.71–3.68), *I*^2^ = 99.0%, *p* < 0.001; pooled RR = 2.50, 95% CI (1.71–3.68), *I*^2^ = 99.0%, *p* < 0.001, respectively (see Table 3). We also conducted a sensitivity analysis in which the Tapiainen et al. paper was excluded due to reporting the probability of a diagnosis of schizophrenia given a dementia diagnosis, in contrast to the other studies included in the meta-analysis. In this analysis, the association between psychotic disorders and dementia was slightly stronger than the main estimate; pooled RR = 2.84, 95% CI (1.84–4.38), *I*^2^ = 99.7%, *p* < 0.001.

**Table 3.** Meta-analysis, subgroup analysis, and sensitivity analyses of the association of non-affective psychotic disorders and dementia

Variable	Number of estimates	*p* value, *I*^2^ (95% CIs)	RR (95% CI)
Main analysis	11	*p* < 0.001, 99.7% (90.5–99.9)	2.52 (1.67–3.80)
Age at onset of psychotic disorder^a^			
<60 years	4	*p* < 0.001, 77.5% (0–94.4)	3.10 (2.33–4.14)
⩾60 years	5	*p* < 0.001, 98.9% (0–99.7)	2.77 (1.74–4.40)
Minimum age (at baseline)^b^			
<60 years	5	*p* < 0.001, 98.9% (0–99.8)	3.16 (1.59–6.28)
⩾60 years	5	*p* < 0.001, 99.8% (45.6–100)	2.61 (1.39–4.92)
Follow-up time			
<10 years	4	*p* < 0.001, 10.9% (0–71.5)	2.64 (1.98–3.51)
⩾10 years	7	*p* < 0.001, 99.8% (93.9–99.9)	2.33 (1.43–3.82)
Geographical region			
Europe	4	*p* < 0.001, 99.5% (0–99.9)	2.04 (1.12–3.71)
Non-Europe	7	*p* < 0.001, 99.4% (65.9–99.9)	2.96 (1.56–5.62)
Study type			
Retrospective	8	*p* < 0.001, 99.8% (91–99.9)	2.34 (1.45–3.78)
Prospective	3	*p* < 0.001, 28.3% (0–81.1)	2.65 (2.10–3.35)
Sex (% female)^c^			
<60	4	*p* < 0.001, 96.7% (0–99.3)	2.10 (1.42–3.12)
⩾60	4	*p* < 0.001, 79.0% (0–94.4)	3.24 (2.09–5.02)
Psychiatric diagnosis			
Schizophrenia	7	*p* < 0.001, 95.9% (0–99.1)	2.13 (1.66–2.73)
Psychotic disorders	4	*p* < 0.001, 99.4% (77.4–99.8)	2.78 (1.57–4.91)
Year of study publication			
Before 2020	6	*p* < 0.001, 95.8% (0–98.8)	1.93 (1.34–2.77)
2020 or after	5	*p* < 0.001, 99.9% (76.1–100)	3.13 (1.55–6.33)
Sensitivity analyses			
Good quality studies	8	*p* < 0.001, 99.1% (85–99.7)	2.50 (1.71–3.68)
Studies that controlled for covariates	8	*p* < 0.001, 99.1% (85–99.7)	2.50 (1.71–3.68)

a Five studies excluded due to insufficient data (Kershenbaum et al., 2020; Kodesh et al., 2020; Richmond-Rakerd et al., 2022; Stroup et al., 2021; Tapiainen et al., 2017).

b One study excluded due to lack of data on minimum age (Tapiainen et al., 2017).

c Three studies excluded due to insufficient data on proportion of females (Ribe et al., 2015; Richmond-Rakerd et al., 2022; Tapiainen et al., 2017).

### Interval between incidence of psychotic disorders and dementia

For factors such as the interval between onset of non-affective psychotic disorders, and dementia diagnosis, and age-at-onset of dementia, there were insufficient data available to conduct further analyses, therefore we describe these narratively. Four studies investigated the interval between onset of a psychotic disorder and a dementia diagnosis and found that the association was stronger when the interval between the two diagnoses was shorter. Almeida et al. (2018) stratified their analysis by those living with a psychotic disorder for <5 years, and ⩾10 years and found that the risk was higher for those living with a psychotic disorder for fewer years. Similarly, Stafford et al. (2021) found that the strongest association was observed in the first few years following a psychotic disorder diagnosis. For both LODD and LOATP, associations were stronger at 0–6 months after receiving a psychotic disorder diagnosis compared to 12+ months (Kørner et al., 2008, 2009a).

### Age-at-onset of dementia

Three studies investigated the effect of average age of dementia diagnosis. All three studies found that individuals with non-affective psychotic disorders were younger at dementia diagnosis, compared to those without (Ribe et al., 2015; Stafford et al., 2021; Stroup et al., 2021). In the study by Stafford et al. (2021) the median age of dementia diagnosis was lower in the VLOSLP group compared to those without VLOSLP. In the Ribe et al. (2015) study, dementia was diagnosed before the age of 65 for 22.4% of people with schizophrenia compared to 6.3% of people without schizophrenia, whereas in the Stroup et al. (2021) study the prevalence of dementia in people with schizophrenia at 66 years was similar to the prevalence of dementia at 88 years for people in the general population.

### Psychotic disorder diagnosis

Two studies focused on psychotic disorder subtypes other than schizophrenia or combined non-affective psychotic disorders (Kørner et al., 2008, 2009a). Both studies on LOATP and LODD reported an increased risk of dementia among people with LOATP or LODD compared to controls (SMR = 8.12, 95% CI (6.7–9.74), and IRR = 5.49, 95% CI (4.81–6.26), respectively).

### Comorbidities

Two studies examined associations between psychotic disorders and dementia in relation to comorbidities (Lin et al., 2018; Ribe et al., 2015). One study found an increased risk of dementia for individuals diagnosed with schizophrenia with comorbid cardiovascular disease: HR = 4.27, 95% CI (3.64–4.99) *v.* no cardiovascular disease HR = 2.36 95% CI (1.45–3.87) (interaction *p* < 0.001). Associations between schizophrenia and dementia were also stronger in the presence of diabetes, hypertension, Parkinson's disease, traumatic head injury, and alcohol-related disorders (all interactions *p* < 0.05). Ribe et al. found that associations were stable in subgroups relating to medical comorbidities, with no evidence of differences in relation to diabetes, ischaemic heart disease, congestive heart failure, atrial fibrillation, or peripheral vascular disease. Schizophrenia showed stronger associations with dementia in those without cerebrovascular disease; IRR = 2.23, 95% CI (2.08–2.39) *v.* IRR = 1.71, 95% CI (1.47–2.00), *p* = 0.002. In contrast to the Lin et al. study, the association between schizophrenia and dementia was stronger in those without comorbid substance abuse; IRR = 1.96, 95% CI (1.82–2.11) *v.* IRR = 1.09, 95% CI (0.95–1.24), *p* < 0.001.

### Anti-psychotic medication

Two studies stratified analyses by anti-psychotic medications (Kodesh et al., 2020; Lin et al., 2018). One study found that both first-generation anti-psychotics (FGAs) and second-generation anti-psychotics (SGAs) were associated with a lower risk of dementia in individuals with psychotic disorders compared to those not taking anti-psychotic medication (Lin et al., 2018). However, in the second study FGAs were associated with a decreased risk of dementia across the whole sample, whereas SGAs were associated with an increased risk (Kodesh et al., 2020).

## Discussion

### Summary of the evidence

To the best of our knowledge, this is the first high-quality systematic review and meta-analysis to synthesise worldwide evidence on the association between non-affective psychotic disorders and future dementia risk. Our comprehensive search identified over 9000 records and included a separate search of the grey literature. Our meta-analysis has shown that the risk of dementia in individuals with non-affective psychotic disorders is 2.52 the risk of those without a non-affective psychotic disorder. This is an important finding indicating that the risk of future dementia attributed to non-affective psychotic disorders is higher than the relative risk associated with other psychiatric disorders and future dementia, such as depression, anxiety, and PTSD (Günak et al., 2020; Kuring, Mathias, & Ward, 2020). Importantly, our results remained robust after excluding lower-quality studies and studies that did not adjust for confounders.

A significant contribution of our review is the finding that the association between psychotic disorders and dementia was present in individuals with a range of non-affective psychotic disorders such as LODD and LOATP, and in those with typical-onset (onset <40 years) as well as late-onset psychotic disorders (onset >40 years), and VLOSLP (onset >60 years). The association was stronger, however, in those with typical and late-onset psychotic disorders, in studies following individuals for <10 years, and in studies conducted in non-European countries. Associations were also stronger in studies with a higher proportion of females and a younger minimum age at baseline. Our narrative synthesis provided some evidence that people with psychotic disorders tended to be younger at dementia diagnosis and that the association between psychotic disorders and dementia was stronger when the interval between the two diagnoses was shorter. Our findings highlight a need for further research examining whether findings vary across different dementia subtypes, for example Alzheimer's disease relative to vascular dementia, and to formally test whether the age-at-onset of dementia differs between those with and without psychotic disorders.

### Mechanisms

There are several potential causal mechanisms that may explain the association between psychotic disorders and increased dementia risk. Schizophrenia has been posited to be a disorder of accelerated ageing, whereby the physiological changes of ageing associated with the disorder may occur earlier than normal, predisposing individuals to cognitive decline (Kirkpatrick, Messias, Harvey, Fernandez-Egea, & Bowie, 2008; Okusaga, 2014). Lower educational attainment and impairments in cognitive functioning which are important markers of psychotic disorders could also lead to increased risk by limiting individuals' cognitive reserve, thereby increasing vulnerability to exhibiting dementia symptoms (Stern, 2012). Psychotic disorders may also confer greater risk via several underlying disease mechanisms such as cardiovascular disease, and obesity, which are known risk factors for both dementia and non-affective psychotic disorders (Cohn, Prud'homme, Streiner, Kameh, & Remington, 2004; Jeste, Gladsjo, Lindamer, & Lacro, 1996; Stahl, Mignon, & Meyer, 2009). People with psychotic disorders are also more likely to have a poor diet, smoke and abuse substances, which are health behaviours that may accelerate neuropathology associated with dementia (Lambert et al., 2005; Mamakou, Thanopoulou, Gonidakis, Tentolouris, & Kontaxakis, 2018). This remains an important area for future investigation, given that only a few of the identified studies investigated the role of comorbidities in the association between psychotic disorders and dementia, and findings were mixed overall. Examination of psychiatric comorbidities, such as depression and PTSD, was particularly lacking.

Furthermore, there is a substantial mortality gap between people with psychotic disorders and the general population (Saha, Chant, & McGrath, 2007). In support of our findings, a recent study which did not meet our inclusion criteria due to ascertaining dementia via cause-of-death data only, demonstrated substantial premature mortality among people with serious mental illness using primary care data, with particularly high standardised mortality ratios identified for conditions including dementia (John et al., 2018).

In addition, it has been posited that antipsychotic medications could contribute to an increased risk of dementia (Jonas, Abi-Dargham, & Kotov, 2021). A nationwide population-based study in South Korea found that, among older people with depressive disorders, users of atypical antipsychotics were at an increased risk of dementia (Kim et al., 2021), and a small trial of first episode psychosis patients found a potential negative role of second-generation antipsychotics (Faber, Smid, Van Gool, Wiersma, & Van Den Bosch, 2012). In contrast, we identified only two studies examining the role of antipsychotic medications, which yielded mixed findings, with one study reporting a protective effect on dementia risk in people with schizophrenia, while another study found a protective effect for FGAs, but an increased risk of dementia in relation to SGAs. Given these mixed findings, this remains an important area for future research.

Although our data indicate an increased risk of dementia in non-affective psychotic disorders, it remains possible that this association could be partly explained by reverse causation, whereby psychotic symptoms could represent markers of prodromal dementia rather than causal risk factors (Ismail et al., 2016). An important finding of our review is that, although subgroup analyses indicated stronger associations in studies with shorter follow-up periods, associations remained substantial in studies with follow-up periods longer than 10 years, and in studies including individuals diagnosed with psychotic disorders in early adulthood and mid-life. This therefore suggests that psychotic disorders could represent a causal risk factor for dementia, given that associations found with longer follow-up periods are less likely to be explained by reverse causation.

In line with this hypothesis, our subgroup analysis suggested that individuals with an earlier onset of psychotic disorders had a greater risk of dementia than individuals diagnosed with VLOSLP. People with typical and late-onset psychotic disorders are likely to have been diagnosed with a psychotic disorder many years before the symptoms of dementia begin, consistent with the possibility that psychotic disorders are a risk factor for dementia. A possible explanation for this finding is that individuals with an earlier onset have a longer exposure to the adverse effects of living with a psychotic disorder, resulting in accumulation of several negative health behaviours and medical comorbidities increasing dementia risk.

Further longitudinal studies with long follow-up periods and studies that model trajectories of association over time are required to elucidate possible mechanisms underlying the relationship between psychotic disorders and increased dementia risk (Singh-Manoux et al., 2017). In addition, triangulation across multiple study designs, including studies involving twin- and sibling-difference designs, would provide further insight into the likelihood of a causal relationship between psychotic disorders and dementia. These studies should also investigate whether factors such as medical comorbidities, health behaviours, substance abuse, educational attainment, and anti-psychotic medication may explain the association between psychotic disorders and future dementia. Understanding which lifestyle risk factors or comorbidities impact the risk of dementia for people with psychotic disorders could help develop treatments better tailored to people living with serious mental illness, helping to reduce or delay number of years living with a neurodegenerative disease.

### Strengths and limitations

We used a comprehensive and highly sensitive search strategy, including a grey literature search of the worldwide evidence. Our meta-analysis included studies from several countries, most of which reported data on large sample sizes and long follow-up periods. Most of our studies were also assessed to be of high quality, which increases our confidence in the conclusions of our review. Our meta-analytic estimates are also similar to those reported in an earlier review examining the association between schizophrenia and risk of dementia (Cai & Huang, 2018). Despite these strengths however, our study has several limitations. First, diagnosing dementia in individuals with psychotic disorders is particularly challenging. Cognitive deficits and behavioural symptoms in psychotic disorders may be misdiagnosed as dementia for some individuals, or in some cases, dementia symptoms could be misattributed as being symptoms of psychosis (Ribe et al., 2015). It is possible therefore, that the over or underestimation of dementia in individual studies could have affected our pooled estimate. Despite our subgroup and sensitivity analyses, heterogeneity remained high, and we could not detect its source. Nonetheless, almost all included studies found evidence of an association between psychotic disorders and dementia, with variation mainly in the magnitude of observed associations.

In addition, there were insufficient data available to conduct formal subgroup analyses for all factors of interest, including interval between psychotic disorder onset and dementia diagnosis, and age-at-onset of dementia, highlighting the need for further longitudinal evidence in this area. Most studies included in our meta-analysis were retrospective register-based studies, which often lack detailed information on socio-demographic confounders (Talari & Goyal, 2020), and may be more prone to detection bias whereby comorbidities could be more frequently diagnosed in people with psychotic disorders due to higher levels of contact with mental health services (Viswanathan, Berkman, Dryden, & Hartling, 2013). However, in contrast, we found slightly stronger associations between psychotic disorders and dementia in prospective studies, relative to retrospective studies. One possibility is that those with psychotic disorders may have poorer access to healthcare services; hence retrospective studies involving medical records may represent a healthier subset of the population with psychotic disorders who may be less likely to have comorbidities, including dementia, than those in prospective studies.

## Conclusion

This systematic review and meta-analysis provides an up-to-date estimate and narrative synthesis of the association between non-affective psychotic disorders and future dementia risk. Our findings indicate that non-affective psychotic disorders constitute an important and potentially modifiable risk factor for all-cause dementia and highlight the need to revise current models of dementia prevention across the life span (Livingston et al., 2020). Individuals with psychotic disorders and people who develop psychotic symptoms later in life require careful monitoring for symptoms of cognitive and functional decline. Given the robust association observed between psychotic disorders and future dementia risk, our findings should be reflected in future clinical guidelines for the treatment and care of people living with non-affective psychotic disorders.

## Data Availability

Data contributing to the findings of this study are available on request from the study authors (SEM and JS); contact at e-mail: j.stafford@ucl.ac.uk.

## Supplementary material

For supplementary material accompanying this paper visit https://doi.org/10.1017/S0033291722002781. click here to view supplementary material

## References

[ref1] Alkan, E., Davies, G., & Evans, S. L. (2021). Cognitive impairment in schizophrenia: Relationships with cortical thickness in fronto-temporal regions, and dissociability from symptom severity. Nature Portfolio Journal Schizophrenia, 7(1), 1–9. doi: 10.1038/s41537-021-00149-0 PMC7973472 33737508

[ref2] Almeida, O. P., Ford, A. H., Hankey, G. J., Yeap, B. B., Golledge, J., & Flicker, L. (2018). Risk of dementia associated with psychotic disorders in later life: The health in men study (HIMS). Psychological Medicine, 49(2), 232–242. doi: 10.1017/S003329171800065X 29564993

[ref3] American Psychiatric Association. (2013). Diagnostic and statistical manual of mental disorders (5th ed.). Arlington, VA: American Psychiatric Association.

[ref4] Arciniegas, D. B. (2015). Psychosis. Continuum: Lifelong Learning in Neurology, 21(3), 715–736. doi: 10.1212/01.CON.0000466662.89908.e7 26039850 PMC4455840

[ref5] Blacker, D., Albert, M. S., Bassett, S. S., Go, R. C., Harrell, L. E., & Folstein, M. F. (1994). Reliability and validity of NINCDS-ADRDA criteria for Alzheimer's disease: The national institute of mental health genetics initiative. Archives of Neurology, 51(12), 1198–1204. doi: 10.1001/archneur.1994.00540240042014 7986174

[ref6] Brodaty, H., Sachdev, P., Koschera, A., Monk, D., & Cullen, B. (2003). Long-term outcome of late-onset schizophrenia: 5-year follow-up study. The British Journal of Psychiatry, 183(3), 213–219. doi: 10.1192/bjp.183.3.213 12948993

[ref7] Cai, L., & Huang, J. (2018). Schizophrenia and risk of dementia: A meta-analysis study. Neuropsychiatric Disease and Treatment, 14, 2047–2055. Retrieved from https://www.ncbi.nlm.nih.gov/pmc/articles/PMC6095111/. 30147318 10.2147/NDT.S172933 PMC6095111

[ref8] Cohn, T., Prud'homme, D., Streiner, D., Kameh, H., & Remington, G. (2004). Characterizing coronary heart disease risk in chronic schizophrenia: High prevalence of the metabolic syndrome. The Canadian Journal of Psychiatry, 49(11), 753–760. doi: 10.1177/070674370404901106 15633853

[ref9] Duong, S., Patel, T., & Chang, F. (2017). Dementia: What pharmacists need to know. Canadian Pharmacists Journal, 150(2), 118–129. doi: 10.1177/1715163517690745 28405256 PMC5384525

[ref11] Faber, G., Smid, H. G. O. M., Van Gool, A. R., Wiersma, D., & Van Den Bosch, R. J. (2012). The effects of guided discontinuation of antipsychotics on neurocognition in first onset psychosis. European Psychiatry, 27(4), 275–280. doi: 10.1016/j.eurpsy.2011.02.003 21561741

[ref12] Ferri, C. P., Prince, M., Brayne, C., Brodaty, H., Fratiglioni, L., Ganguli, M., … Scazufca, M. (2005). Global prevalence of dementia: A Delphi consensus study. The Lancet, 366(9503), 2112–2117. doi: 10.1016/S0140-6736(05)67889-0 PMC2850264 16360788

[ref13] Greenland, S. (1987). Quantitative methods in the review of epidemiologic literature. Epidemiologic Reviews, 9(1), 1–30. doi: 10.1093/oxfordjournals.epirev.a036298 3678409

[ref14] Günak, M. M., Billings, J., Carratu, E., Marchant, N. L., Favarato, G., & Orgeta, V. (2020). Post-traumatic stress disorder as a risk factor for dementia: Systematic review and meta-analysis. The British Journal of Psychiatry, 217(5), 600–608. doi: 10.1192/bjp.2020.150 32933591

[ref15] Higgins, J. P., Thomas, J., Chandler, J., Cumpston, M., Li, T., Page, M. J., … Welch, V. A. (Eds.). (2019). Cochrane handbook for systematic reviews of interventions. Hoboken, NJ: Wiley-Blackwell.

[ref16] Howard, R., Rabins, P. V., Seeman, M. V., & Jeste, D. V., & the International Late-Onset Schizophrenia Group. (2000). Late-onset schizophrenia and very-late-onset schizophrenia-like psychosis: An international consensus. American Journal of Psychiatry, 157(2), 172–178. doi: 10.1176/appi.ajp.157.2.172 10671383

[ref17] Ioannidis, J. P., & Trikalinos, T. A. (2007). The appropriateness of asymmetry tests for publication bias in meta-analyses: A large survey. Canadian Medical Association Journal, 176(8), 1091–1096. doi: 10.1503/cmaj.060410 17420491 PMC1839799

[ref18] Ismail, Z., Smith, E. E., Geda, Y., Sultzer, D., Brodaty, H., Smith, G., … Lyketsos, C. G. (2016). Neuropsychiatric symptoms as early manifestations of emergent dementia: Provisional diagnostic criteria for mild behavioral impairment. Alzheimer's & Dementia, 12(2), 195–202. doi: 10.1016/j.jalz.2015.05.017 PMC4684483 26096665

[ref19] Jeste, D. V., Gladsjo, J. A., Lindamer, L. A., & Lacro, J. P. (1996). Medical comorbidity in schizophrenia. Schizophrenia Bulletin, 22(3), 413–430. doi: 10.1093/schbul/22.3.413 8873293

[ref20] John, A., McGregor, J., Jones, I., Lee, S. C., Walters, J. T., Owen, M. J., … Lloyd, K. (2018). Premature mortality among people with severe mental illness – New evidence from linked primary care data. Schizophrenia Research, 199, 154–162. doi: 10.1016/j.schres.2018.04.009 29728293

[ref21] Jonas, K., Abi-Dargham, A., & Kotov, R. (2021). Two hypotheses on the high incidence of dementia in psychotic disorders. JAMA psychiatry, 78(12), 1305–1306. doi: 10.1001/jamapsychiatry.2021.2584 34524413 PMC10805107

[ref22] Kahn, R. S. (2019). On the specificity of continuous cognitive decline in schizophrenia. The American Journal of Psychiatry, 176(10), 774–776. doi: 10.1176/appi.ajp.2019.19080794 31569987

[ref23] Kershenbaum, A., Cardinal, R. N., Chen, S., Underwood, B. R., Seyedsalehi, A., Lewis, J., … Rubinsztein, J. S. (2020). Investigation of risk of dementia diagnosis and death in patients in older people's secondary care mental health services. International Journal of Geriatric Psychiatry, 36(4), 573–582. doi: 10.1002/gps.5455 33113255 PMC7984055

[ref24] Kessler, R. C., Amminger, G. P., Aguilar-Gaxiola, S., Alonso, J., Lee, S., & Ustün, T. B. (2007). Age of onset of mental disorders: A review of recent literature. Current Opinion in Psychiatry, 20(4), 359–364. doi: 10.1097/YCO.0b013e32816ebc8c 17551351 PMC1925038

[ref25] Kim, J., Ha, T. H., Kim, K., Lee, E. M., Kim, H., Kim, D. K., … Myung, W. (2021). Atypical antipsychotics augmentation in patients with depressive disorder and risk of subsequent dementia: A nationwide population-based cohort study. Journal of Alzheimer's Disease, 80(1), 197–207. doi: 10.3233/JAD-200994 33523000

[ref26] Kirkpatrick, B., Messias, E., Harvey, P. D., Fernandez-Egea, E., & Bowie, C. R. (2008). Is schizophrenia a syndrome of accelerated aging? Schizophrenia Bulletin, 34(6), 1024–1032. doi: 10.1093/schbul/sbm140 18156637 PMC2632500

[ref27] Kodesh, A., Goldberg, Y., Rotstein, A., Weinstein, G., Reichenberg, A., Sandin, S., & Levine, S. Z. (2020). Risk of dementia and death in very-late-onset schizophrenia-like psychosis: A national cohort study. Schizophrenia Research, 223, 220–226. doi: 10.1016/j.schres.2020.07.020 32807646

[ref28] Kørner, A., Lopez, A. G., Lauritzen, L., Andersen, P. K., & Kessing, L. V. (2008). Delusional disorder in old age and the risk of developing dementia – A nationwide register-based study. Aging and Mental Health, 12(5), 625–629. doi: 10.1080/13607860802343118 18855178

[ref29] Kørner, A., Lopez, A. G., Lauritzen, L., Andersen, P. K., & Kessing, L. V. (2009a). Acute and transient psychosis in old age and the subsequent risk of dementia: A nationwide register-based study. Geriatrics & Gerontology International, 9(1), 62–68. doi: 10.1111/j.1447-0594.2009.00505.x 19260981

[ref30] Kørner, A., Lopez, A. G., Lauritzen, L., Andersen, P. K., & Kessing, L. V. (2009b). Late and very-late first-contact schizophrenia and the risk of dementia – A nationwide register based study. International Journal of Geriatric Psychiatry, 24(1), 61–67. doi: 10.1002/gps.2075 18561206

[ref31] Kuring, J. K., Mathias, J. L., & Ward, L. (2020). Risk of dementia in persons who have previously experienced clinically-significant depression, anxiety, or PTSD: A systematic review and meta-analysis. Journal of Affective Disorders, 274, 247–261. doi: 10.1016/j.jad.2020.05.020 32469813

[ref32] Lambert, M., Conus, P., Lubman, D. I., Wade, D., Yuen, H., Moritz, S., … Schimmelmann, B. G. (2005). The impact of substance use disorders on clinical outcome in 643 patients with first-episode psychosis. Acta Psychiatrica Scandinavica, 112(2), 141–148. doi: 10.1111/j.1600-0447.2005.00554.x 15992396

[ref33] Lin, C. E., Chung, C. H., Chen, L. F., & Chi, M. J. (2018). Increased risk of dementia in patients with schizophrenia: A population-based cohort study in Taiwan. European Psychiatry, 53, 7–16. doi: 10.1016/j.eurpsy.2018.05.005 29859379

[ref34] Lin, L., & Chu, H. (2018). Quantifying publication bias in meta-analysis. Biometrics, 74(3), 785–794. doi: 10.1111/biom.12817 29141096 PMC5953768

[ref35] Livingston, G., Huntley, J., Sommerlad, A., Ames, D., Ballard, C., Banerjee, S., … Mukadam, N. (2020). Dementia prevention, intervention, and care: 2020 report of the Lancet Commission. The Lancet, 396(10248), 413–446. doi: 10.1016/S0140-6736(20)30367-6 PMC7392084 32738937

[ref36] Mamakou, V., Thanopoulou, A., Gonidakis, F., Tentolouris, N., & Kontaxakis, V. (2018). Schizophrenia and type 2 diabetes mellitus. Psychiatrike, 29(1), 64–73. doi: 10.22365/jpsych.2018.291.64 29754122

[ref37] Okusaga, O. O. (2014). Accelerated aging in schizophrenia patients: The potential role of oxidative stress. Aging and Disease, 5(4), 256. doi: 10.14336/AD.2014.0500256 25110609 PMC4113515

[ref38] Page, M. J., McKenzie, J. E., Bossuyt, P. M., Boutron, I., Hoffmann, T. C., Mulrow, C. D., … Moher, D. (2021). The PRISMA 2020 statement: An updated guideline for reporting systematic reviews. Systematic Reviews, 10(1), 1–11. doi: 10.1186/s13643-021-01626-4 33781348 PMC8008539

[ref39] Prince, M. J., Wimo, A., Guerchet, M. M., Ali, G. C., Wu, Y. T., & Prina, M. (2015). *World Alzheimer Report* 2015 *– The Global Impact of Dementia: An analysis of prevalence, incidence, cost and trends*. Retrieved from https://www.alz.co.uk/research/WorldAlzheimerReport2015.pdf.

[ref40] Ribe, A. R., Laursen, T. M., Charles, M., Katon, W., Fenger-Grøn, M., Davydow, D., … Vestergaard, M. (2015). Long-term risk of dementia in persons with schizophrenia: A Danish population-based cohort study. JAMA Psychiatry, 72(11), 1095–1101. doi: 10.1001/jamapsychiatry.2015.1546 26444987

[ref41] Richmond-Rakerd, L. S., D'Souza, S., Milne, B. J., Caspi, A., & Moffitt, T. E. (2022). Longitudinal associations of mental disorders with dementia: 30-year analysis of 1.7 million New Zealand citizens. JAMA Psychiatry, 79(4), 333–340. doi: 10.1001/jamapsychiatry.2021.4377 35171209 PMC8851362

[ref42] Saha, S., Chant, D., & McGrath, J. (2007). A systematic review of mortality in schizophrenia: Is the differential mortality gap worsening over time? Archives of General Psychiatry, 64(10), 1123–1131. doi: 10.1001/archpsyc.64.10.1123 17909124

[ref43] Singh-Manoux, A., Dugravot, A., Fournier, A., Abell, J., Ebmeier, K., Kivimäki, M., & Sabia, S. (2017). Trajectories of depressive symptoms before diagnosis of dementia: A 28-year follow-up study. JAMA Psychiatry, 74(7), 712–718. doi: 10.1001/jamapsychiatry.2017.0660 28514478 PMC5710246

[ref44] Stafford, J., Dykxhoorn, J., Sommerlad, A., Dalman, C., Kirkbride, J. B., & Howard, R. (2021). Association between risk of dementia and very late-onset schizophrenia-like psychosis: A Swedish population-based cohort study. Psychological Medicine, 1–9. doi: 10.1017/S0033291721002099 PMC9975996 34030750

[ref45] Stafford, J., Howard, R., Dalman, C., & Kirkbride, J. B. (2019). The incidence of nonaffective, nonorganic psychotic disorders in older people: A population-based cohort study of 3 million people in Sweden. Schizophrenia Bulletin, 45(5), 1152–1160. doi: 10.1093/schbul/sby147 30339239 PMC6737541

[ref46] Stafford, J., Howard, R., & Kirkbride, J. B. (2018). The incidence of very late-onset psychotic disorders: A systematic review and meta-analysis, 1960–2016. Psychological Medicine, 48(11), 1775–1786. doi: 10.1017/S0033291717003452 29198197

[ref47] Stahl, S. M., Mignon, L., & Meyer, J. M. (2009). Which comes first: Atypical antipsychotic treatment or cardiometabolic risk? Acta Psychiatrica Scandinavica, 119(3), 171–179. doi: 10.1111/j.1600-0447.2008.01334.x 19178394

[ref48] Stang, A. (2010). Critical evaluation of the Newcastle–Ottawa scale for the assessment of the quality of nonrandomized studies in meta-analyses. European Journal of Epidemiology, 25(9), 603–605. doi: 10.1007/s10654-010-9491-z 20652370

[ref49] Stern, Y. (2012). Cognitive reserve in ageing and Alzheimer's disease. The Lancet Neurology, 11(11), 1006–1012. doi: 10.1016/S1474-4422(12)70191-6 23079557 PMC3507991

[ref50] Stroup, T. S., Olfson, M., Huang, C., Wall, M. M., Goldberg, T., Devanand, D. P., & Gerhard, T. (2021). Age-specific prevalence and incidence of dementia diagnoses among older US adults with schizophrenia. JAMA Psychiatry, 78(6), 632–641. doi: 10.1001/jamapsychiatry.2021.0042 33688938 PMC7948106

[ref51] Talari, K., & Goyal, M. (2020). Retrospective studies – Utility and caveats. Journal of the Royal College of Physicians of Edinburgh, 50(4), 398–402. doi: 10.4997/JRCPE.2020.409 33469615

[ref52] Tapiainen, V., Hartikainen, S., Taipale, H., Tiihonen, J., & Tolppanen, A. M. (2017). Hospital-treated mental and behavioral disorders and risk of Alzheimer's disease: A nationwide nested case-control study. European Psychiatry, 43, 92–98. doi: 10.1016/j.eurpsy.2017.02.486 28388490

[ref53] Vahia, I. V., Palmer, B. W., Depp, C., Fellows, I., Golshan, S., Kraemer, H. C., & Jeste, D. V. (2010). Is late-onset schizophrenia a subtype of schizophrenia? Acta Psychiatrica Scandinavica, 122(5), 414–426. doi: 10.1111/j.1600-0447.2010.01552.x 20199491 PMC3939834

[ref54] Viswanathan, M., Berkman, N. D., Dryden, D. M., & Hartling, L. (2013). Approaches to assessing the risk of bias in studies. In Assessing risk of bias and confounding in observational studies of interventions or exposures: Further development of the RTI item bank. United States: Agency for Healthcare Research and Quality. 24006553

[ref55] Wimo, A., Guerchet, M., Ali, G. C., Wu, Y. T., Prina, A. M., Winblad, B., … Prince, M. (2017). The worldwide costs of dementia 2015 and comparisons with 2010. Alzheimer's and Dementia, 13(1), 1–7. 10.1016/j.jalz.2016.07.150 PMC5232417 27583652

[ref56] Wise, E. A., Rosenberg, P. B., Lyketsos, C. G., & Leoutsakos, J. M. (2019). Time course of neuropsychiatric symptoms and cognitive diagnosis in National Alzheimer's Coordinating Centers volunteers. Alzheimer's & Dementia, 11, 333–339. doi: 10.1016/j.dadm.2019.02.006 PMC6476801 31024987

[ref57] World Health Organization. (2017). Global action plan on the public health response to dementia, *2017*–*2025*. Geneva: World Health Organization. Retrieved from https://apps.who.int/iris/bitstream/handle/10665/259615/?sequence=1.

[ref58] World Health Organization. (2018). International classification of diseases for mortality and morbidity statistics *(11*th Revision*)*. Geneva: World Health Organization. Retrieved from https://icd.who.int/browse11/l-m/en.

