# Supplementary material for: Non-affective psychotic disorders and risk of dementia: a systematic review and meta-analysis
Source: Psychol Med. 2022 Oct 6;52(15):3323–35. doi: 10.1017/S0033291722002781 (PMC9772917; doi:10.1017/S0033291722002781)
Supplement: Supplementary file 1 [file S0033291722002781sup001.docx]

**Supplementary Material**

**Supplementary Table 1.**

| **Section and Topic** | **Item #** | **Checklist item** | **Location where item is reported** |
| --- | --- | --- | --- |
| **TITLE** | | |  |
| Title | 1 | Identify the report as a systematic review. | Page 1 |
| **ABSTRACT** | | |  |
| Abstract | 2 | See the PRISMA 2020 for Abstracts checklist. | Page 2 |
| **INTRODUCTION** | | |  |
| Rationale | 3 | Describe the rationale for the review in the context of existing knowledge. | Pages 4-6 |
| Objectives | 4 | Provide an explicit statement of the objective(s) or question(s) the review addresses. | Page 6 |
| **METHODS** | | |  |
| Eligibility criteria | 5 | Specify the inclusion and exclusion criteria for the review and how studies were grouped for the syntheses. | Page 7 |
| Information sources | 6 | Specify all databases, registers, websites, organisations, reference lists and other sources searched or consulted to identify studies. Specify the date when each source was last searched or consulted. | Pages 6-7 |
| Search strategy | 7 | Present the full search strategies for all databases, registers and websites, including any filters and limits used. | Supplementary material |
| Selection process | 8 | Specify the methods used to decide whether a study met the inclusion criteria of the review, including how many reviewers screened each record and each report retrieved, whether they worked independently, and if applicable, details of automation tools used in the process. | Page 7 |
| Data collection process | 9 | Specify the methods used to collect data from reports, including how many reviewers collected data from each report, whether they worked independently, any processes for obtaining or confirming data from study investigators, and if applicable, details of automation tools used in the process. | Page 7 |
| Data items | 10a | List and define all outcomes for which data were sought. Specify whether all results that were compatible with each outcome domain in each study were sought (e.g. for all measures, time points, analyses), and if not, the methods used to decide which results to collect. | Page 7-8 |
|  | 10b | List and define all other variables for which data were sought (e.g. participant and intervention characteristics, funding sources). Describe any assumptions made about any missing or unclear information. | Page 7-8 |
| Study risk of bias assessment | 11 | Specify the methods used to assess risk of bias in the included studies, including details of the tool(s) used, how many reviewers assessed each study and whether they worked independently, and if applicable, details of automation tools used in the process. | Page 8 |
| Effect measures | 12 | Specify for each outcome the effect measure(s) (e.g. risk ratio, mean difference) used in the synthesis or presentation of results. | Pages 7-8 |
| Synthesis methods | 13a | Describe the processes used to decide which studies were eligible for each synthesis (e.g. tabulating the study intervention characteristics and comparing against the planned groups for each synthesis (item #5)). | Page 8 |
|  | 13b | Describe any methods required to prepare the data for presentation or synthesis, such as handling of missing summary statistics, or data conversions. | Page 7-8 |
|  | 13c | Describe any methods used to tabulate or visually display results of individual studies and syntheses. | Page 7-8 |
|  | 13d | Describe any methods used to synthesize results and provide a rationale for the choice(s). If meta-analysis was performed, describe the model(s), method(s) to identify the presence and extent of statistical heterogeneity, and software package(s) used. | Page 8 |
|  | 13e | Describe any methods used to explore possible causes of heterogeneity among study results (e.g. subgroup analysis, meta-regression). | Page 8 |
|  | 13f | Describe any sensitivity analyses conducted to assess robustness of the synthesized results. | Page 8 |
| Reporting bias assessment | 14 | Describe any methods used to assess risk of bias due to missing results in a synthesis (arising from reporting biases). | Page 8 |
| Certainty assessment | 15 | Describe any methods used to assess certainty (or confidence) in the body of evidence for an outcome. | Page 7-8 |
| **RESULTS** | | |  |
| Study selection | 16a | Describe the results of the search and selection process, from the number of records identified in the search to the number of studies included in the review, ideally using a flow diagram. | Pages 8-9; Figure 1 |
|  | 16b | Cite studies that might appear to meet the inclusion criteria, but which were excluded, and explain why they were excluded. | Supplementary material |
| Study characteristics | 17 | Cite each included study and present its characteristics. | Page 8-9; Table 1 |
| Risk of bias in studies | 18 | Present assessments of risk of bias for each included study. | Page 9; Tables 1 and 2 |
| Results of individual studies | 19 | For all outcomes, present, for each study: (a) summary statistics for each group (where appropriate) and (b) an effect estimate and its precision (e.g. confidence/credible interval), ideally using structured tables or plots. | Pages 9-11 |
| Results of syntheses | 20a | For each synthesis, briefly summarise the characteristics and risk of bias among contributing studies. | Pages 9-11 |
|  | 20b | Present results of all statistical syntheses conducted. If meta-analysis was done, present for each the summary estimate and its precision (e.g. confidence/credible interval) and measures of statistical heterogeneity. If comparing groups, describe the direction of the effect. | Pages 9-13 |
|  | 20c | Present results of all investigations of possible causes of heterogeneity among study results. | Pages 11-13 |
|  | 20d | Present results of all sensitivity analyses conducted to assess the robustness of the synthesized results. | Page 13 |
| Reporting biases | 21 | Present assessments of risk of bias due to missing results (arising from reporting biases) for each synthesis assessed. | Page 13 |
| Certainty of evidence | 22 | Present assessments of certainty (or confidence) in the body of evidence for each outcome assessed. | Pages 9-13 |
| **DISCUSSION** | | |  |
| Discussion | 23a | Provide a general interpretation of the results in the context of other evidence. | Pages 15-16 |
|  | 23b | Discuss any limitations of the evidence included in the review. | Page 19-20 |
|  | 23c | Discuss any limitations of the review processes used. | Page 19-20 |
|  | 23d | Discuss implications of the results for practice, policy, and future research. | Pages 15-21 |
| **OTHER INFORMATION** | | |  |
| Registration and protocol | 24a | Provide registration information for the review, including register name and registration number, or state that the review was not registered. | Page 3 |
|  | 24b | Indicate where the review protocol can be accessed, or state that a protocol was not prepared. | Page 3 |
|  | 24c | Describe and explain any amendments to information provided at registration or in the protocol. | N/A |
| Support | 25 | Describe sources of financial or non-financial support for the review, and the role of the funders or sponsors in the review. | Page 22 |
| Competing interests | 26 | Declare any competing interests of review authors. | Page 3 |
| Availability of data, code and other materials | 27 | Report which of the following are publicly available and where they can be found: template data collection forms; data extracted from included studies; data used for all analyses; analytic code; any other materials used in the review. | Page 23 |

*From:*  Page MJ, McKenzie JE, Bossuyt PM, Boutron I, Hoffmann TC, Mulrow CD, et al. The PRISMA 2020 statement: an updated guideline for reporting systematic reviews. BMJ 2021;372:n71. doi: 10.1136/bmj.n71

For more information, visit: <http://www.prisma-statement.org/>

| **Search terms** |
| --- |
| 1. Psychotic disorders/ 2. Schizophrenia/or schizophrenia, catatonic/ or schizophrenia, disorganized/ or schizophrenia, paranoid/ 3. (schizoaffective or schizophreniform or “delusional disorder” or “late paraphrenia” or “dementia praecox” or “schizophrenia-like psychosis” or “acute and transient psychotic disorder” or “substance-induced psychosis”) .mp. 4. schizophreni*.mp. 5. psychotic or psychosis or psychoses*.mp. 6. dementia/ or alzheimer disease/ or dementia, vascular/ or frontotemporal lobar degeneration/ or lewy body disease/ 7. (dement* or “lewy bodies” or alzhemer* or “frontotemporal dementia” or “vascular dementia” or “multiinfarct dementia” or “presenile dementia” or “senile dementia”).m.p. 8. Cohort studies/ or follow-up studies/ or longitudinal studies/ or prospective studies/ or retrospective studies 9. (Cohort or longitudinal or prospective or retrospective or follow-up or “nested case-control” or “case-cohort”).mp. 10. Risk factors/ 11. (Risk* or “risk factor*”).mp. 12. 1 or 2 or 3 or 4 or 5 13. 6 or 7 14. 8 or 9 or 10 or 11 15. 12 and 13 and 14 16. Limit to English language 17. Limit to Humans |

**Supplementary Table 2. Search strategy for Medline**

**Supplementary Table 3. Modified New-Castle Ottawa Scale for assessing study quality.^32^**

*Modified Newcastle-Ottawa Assessment and Scoring*

| **Selection** |  |
| --- | --- |
| 1. Representativeness of the exposed cohort |  |
| a) Truly representative (1 point)  b) Somewhat representative (1 point)  c) Selected group of users (0 points)  d) No description of the derivation of the cohort (0 points) |  |
| 1. Selection of the non-exposed cohort |  |
| a) Drawn from the same community as the exposed cohort (1 point)  b) Drawn from a different source (0 points)  c) No description of the derivation of the non-exposed cohort (0 points) |  |
| 1. Ascertainment of exposure |  |
| a) Secure record (e.g., medical records) (1 point)  b) Structured interview (1 point)  c) Written self-report (0 points)  d) No description (0 points)  e) Other (0 points) |  |
| 1. Demonstration that outcome of interest was not present at start of the study |  |
| a) Yes (1 point)  b) No (0 points) |  |
| **Comparability and Design^†^** |  |
| 1. Comparability of cohorts on the basis of the design or analysis |  |
| a) Study controls for two or more covariates (1 point)  b) Study controls for less than two covariates (0 points) |  |
| 1. Longitudinal study design |  |
| a) Prospective longitudinal/cohort study (1 point)  b) Retrospective longitudinal/cohort study (0 points) |  |
| **Outcome** |  |
| 1. Assessment of outcome |  |
| a) Independent blind assessment (1 point)  b) Record linkage (1 point)  c) Self-report (0 points)  d) No description (0 points)  e) Other (0 points) |  |
| 1. Was follow-up long enough for outcome to occur (at least 5 years) |  |
| a) Yes (1 point)  b) No (0 points) |  |
| 1. Adequacy of follow-up of cohorts | |
| a) Complete follow-up – all subjects accounted for (1 point)  b) Subjects lost to follow-up unlikely to introduce bias – number lost less than or equal to 20%, or description provided for those lost (1 point)  c) Follow-up rate less than 80% and no description of those lost (0 points)  d) No statement (0 points) | |

*Note:* Good quality: 3 or 4 points in selection domain AND 1 or 2 points in comparability domain AND 2 or 3 stars in outcome domain

Fair quality: 2 points in selection domain AND 1 or 2 points in comparability domain AND 2 or 3 points in outcome domain. Poor quality: 0 or 1 point in selection domain OR 0 points in comparability domain OR 0 or 1 points in outcome domain

This version was taken (with permission) from a systematic review conducted by Gunak et al. (35)

^†^ Modified for this systematic review; studies must control for at least two covariates in order to score a point for control of confounders.

**Supplementary Table 4. Excluded studies with reasons**

| **Study** | **Reason for exclusion** |
| --- | --- |
| Zilkens et al. (2014) | Case- control study |
| Wilcox et al. (2005) | Not primary research |
| Yassa et al. (1993) | Cross-sectional study |
| Talaslahti et al. (2015) | Dementia was not measured as outcome |
| Soares et al. (2017) | Psychotic symptoms not meeting clinical criteria of psychotic disorder diagnoses |
| Shao et al. (2021) | Psychiatric symptoms not meeting diagnosis of psychotic disorders |
| Shah et al. (2012) | Not primary research |
| Radhakrishnan et al. (2012) | Not primary research |
| Porsteinsson et al. (2015) | Not primary research |
| Peters et al. (2015) | Includes people with dementia at baseline |
| Palmer et al. (2003) | Assessed cognitive decline rather than dementia |
| Ostling et al. (2007) | Subthreshold psychotic symptoms |
| O’Brien (2005) | Not primary research |
| Nicolas et al. (2014) | No general population control group |
| San Roman Uria et al. (2016) | Neuroimaging study, not longitudinal |
| Ryu et al. (2015) | Does not assess incident dementia, no control group |
| Rorsman et al. (1985) | No control group |
| Rohde et al. (2016) | Irrelevant (dementia incidence not measured) |
| Nagendra & Snowdon (2020) | Irrelevant, does not compare incidence of dementia in people with delusional disorder to controls |
| Mulsant et al. (1993) | No general population control group |
| Lyketsos & Peters (2015) | Not primary research |
| Savla et al. (2006) | Investigated cognitive performance, not dementia |
| Jorgensen & Munk-Jordensen (1985) | No comparison group |
| Jobe & Harrow (2010) | Not primary research |
| Jeste et al. (1997) | Not primary research |
| Lee & Kennedy (2018) | Not primary research |
| Japlensky (1986) | Not primary research |
| Hymas et al. (1989) | Assesses cognitive decline, not dementia |
| Hybles & Blazer (2003) | Not primary research |
| Hendrie et al. (2014) | Irrelevant; doesn’t examine incidence of dementia |
| Neufeld & O’Rourke (2006) | Not primary research |
| Harvey et al. (1995) | Cognitive decline measured, not dementia |
| Harvey et al. (1999) | Baseline cognitive impairment present, also cognitive decline was measured, not dementia diagnosis. |
| Harvey et al. (1996) | No comparison group |
| La Salvia & Chemali (2011) | Not primary research |
| Hanssen et al (2015) | Cross-sectional study |
| Goldberg et al. (1993) | Cross-sectional study |
| Gerhard et al. (2020) | Cross sectional study + Review |
| Friedman et al. (2001) | Focused on cognitive decline rather than incident dementia |
| Leinonen et al. (2004) | Does not include general population control group |
| Riecher-Rossler et al (1998) | Not primary research |
| Kirkpatrick et al. (2008) | Not primary research |
| Kohler et al. (2013) | Psychotic symptoms, not diagnosis of psychotic disorder |
| Harvey et al. (2005) | Not primary research |
| Calabrese & Corrigan (2005) | Not primary research |
| Beydoun et al. (2015) | Irrelevant, focuses on comorbidities, rather than psychotic disorders and subsequent dementia |
| Cohen & Murante (2018) | Focused on cognitive decline, not schizophrenia |
| Copeland et al (1998) | Does not compare dementia incidence in schizophrenia compared to general population comparison group |
| Hassett (2002) | Not primary research |
| Harvey et al. (2003) | Cognitive decline, not dementia measured |
| Edwin et al. (2021) | Participants had dementia at baseline |
| Diesfeldt & Troost (1995) | Single case-study |
| De Vries et al (2001) | No comparison group |
| Folsom et al. (2009) | Cross-sectional study |
| Fischer & Aguera-Oritz (2018) | Not primary research |
| Arnold & Trojanowski (1996) | Not Primary research |
| Arif (2014) | Not primary research |
| Allegri et al. (2018) | No general population control group |
| Cooper & Holmes (1998) | Case-control study |
| Cohen et al. (2017) | Doesn’t focus on dementia incidence |
| Addonizio (1995) | Not primary research |
| Cort et al. (2018) | Not primary research |
| Rajji, (2017) | Not primary research |
| Jablensky (1986) | Not primary research |
| Chalita et al. (2006) | Case-series with no control group |
| Ahearn et al. (2020) | Special population (veterans) |
| Andreasen et al. (2010) | Not primary research |
| Bridge et al. (1978) | Not primary research |
| Broadway & Mintzer (2007) | Dementia incidence not measured |
| Chemerinski et al. (2006) | No control group |
| Cipriani et al. (2020) | Not primary research |
| DeLisi (2008) | Not primary research |
| Dietlin et al. (2019) | Mild cognitive impairment at baseline |
| Ford et al. (2019) | Case-control study |
| Girard et al. (2011) | No control group |
| Kastrup (1985) | Cross-sectional study |
| Kochunov et al. (2021) | Not relevant; Does not measure incident dementia |
| Liao et al. (2020) | No control group |
| Matsuoka et al. (2015) | Does not measure incident dementia |
| Ostling et al. (2009) | Does not measure incident dementia |
| Rabins & Lavrisha (2003) | Does not measure incident dementia |
| Rochoy et al. (2019) | Uses a machine learning approach |
| Rubin et al. (1988) | Dementia present at baseline |
| Strauss et al. (1981) | Not primary research |
| Urfer-Parnas et al. (2010) | Not primary research |

**Supplementary Table 5. Correlation between potential moderators**

| **Variable and correlation coefficient** | **Minimum baseline age** | **Follow-up time** | **Geographical region** | **Study design** | **Sex** | **Psychiatric diagnosis** | **Year of study publication** |
| --- | --- | --- | --- | --- | --- | --- | --- |
| **Minimum baseline age** | 1.00 |  |  |  |  |  |  |
| **Follow-up time** | 0.00 | 1.00 |  |  |  |  |  |
| **Geographical region** | -0.22 | 0.18 | 1.00 |  |  |  |  |
| **Study design** | -0.22 | 0.39 | 0.46 | 1.00 |  |  |  |
| **Sex** | -0.26 | -0.5 | 0.58 | -0.26 | 1.00 |  |  |
| **Psychiatric diagnosis** | 0.22 | 0.57 | 0.21 | 0.04 | 0.00 | 1.00 |  |
| **Year of study publication** | 0.6 | -0.07 | -0.31 | 0.15 | 0.00 | 0.07 | 1.00 |

**Supplementary Table 6. Meta-regression analysis**

| **Variable** | **Number of estimates** | **Meta-regression RR, p-value, 95% CI** |
| --- | --- | --- |
| **Follow up time**  <10 years  ≥10 years | 4  7 | 0.79, p=0.59, [0.30-2.06] |
| **Geographical region**  Europe  Non-Europe | 4  7 | 0.72, p=0.41, [0.30-1.70] |
| **Psychiatric diagnosis**  Schizophrenia  Psychotic Disorders | 7  4 | 1.20, p=0.66, [0.49-2.91] |
| **Year of study publication**  Before 2020 2020 or after | 6  5 | 1.59, p=0.22, [0.71-3.54] |

**Supplementary Figure 1. Funnel plot**

**Note*: Brodaty et al. (2003) excluded from funnel plot (given that it is an outlier that impacted the scale of the plot)
